# Modifizierter Gluteus-maximus-Transfer zur Therapie der glutealen Insuffizienz

**DOI:** 10.1007/s00064-024-00860-y

**Published:** 2024-08-22

**Authors:** Alexander Zimmerer, Lars Nonnemacher, Maximilian Fischer, Sebastian Gebhardt, André Hofer, Johannes Reichert, Georgi Wassilew

**Affiliations:** 1 Klinik und Poliklinik für Orthopädie und Orthopädische Chirurgie, Universitätsmedizin Greifswald, Ferdinand-Sauerbruch-Str., 17475 Greifswald, Deutschland; 2 Orthopädische Klinik Paulinenhilfe, Stuttgart, Deutschland; 3 Orthopädie und Unfallchirurgie, Elisabeth-Klinik, Olsberg, Deutschland

**Keywords:** Hüftgelenk, Gluteale Insuffizienz, Glutealregion, Trendelenburg, Peritrochantäres Schmerzsyndrom, Hip joint, Abductor deficiency, Gluteal region, Greater trochanteric pain syndrome, Trendelenburg

## Abstract

**Operationsziel:**

Durchführung eines Transfers des M. gluteus maximus mit Refixation am Trochanter major zur Therapie der glutealen Insuffizienz.

**Indikationen:**

Symptomatische gluteale Insuffizienz mit Atrophie und fettiger Degeneration der Glutealmuskeln > 50 % (Grad 3 nach Quartile) bei guter Kraftentwicklung des M. gluteus maximus.

**Kontraindikationen:**

Geringe Atrophie oder fettige Degeneration von weniger als 50 % der Glutealmuskulatur, eingeschränkte Kraft des M. gluteus maximus, Infektionen.

**Operationstechnik:**

Zunächst wird die Fascia lata dorsal des M. tensor fasciae latae inzidiert, wobei die Inzision bis ca. 1,5 cm proximal des Beckenkammes reicht. Eine zweite Inzision halbiert den M. gluteus maximus in Längsrichtung der Muskelfasern und wird in Richtung der Fascia lata nach distal des Trochanter major fortgesetzt. Durch die Inzisionen resultiert ein dreieckiger Muskellappen, welcher angehoben und in einen vorderen und einen hinteren Teil geteilt wird. Der posteriore Lappen wird über den Schenkelhals nach ventral gelegt und an der vorderen Kapsel und vorderen Kante des Trochanter major fixiert. Der anteriore Lappen wird direkt auf das proximale Femur platziert. Hierfür wird mit einer Kugelfräse eine Rinne im Bereich des proximalen Femurs präpariert, um den künftigen Footprint anzufrischen. Der anteriore Lappen wird von der Spitze des Trochanter major in Richtung des Ansatzes des M. vastus lateralis positioniert. Anschließend wird der anteriore Lappen mit transossären Nähten an der geschaffenen Rinne und unter dem angehobenen M. vastus lateralis in 15° Abduktion des Beines fixiert. Zur zusätzlichen Stabilisierung des sehnigen Teils des anterioren Lappens wird distal des Trochanter major eine Schraube eingebracht. Der M. vastus lateralis wird an der distalen Spitze des anterioren Lappens befestigt, und der verbleibende M. gluteus maximus wird mit der Fascia lata vernäht, um den anterioren Lappen abzudecken. Ergänzend kann ein Lappen des M. tensor fasciae latae mobilisiert und auf die Rekonstruktion adaptiert werden. Schichtweiser Wundverschluss.

**Ergebnisse:**

Die gezeigte Technik eines M.-gluteus-maximus-Transfers stellt eine Methode zur Behandlung chronischer glutealer Insuffizienzen dar und verbessert in kurzzeitigen Follow-ups die Abduktionsfunktion sowie das Gangbild. Es wurden 15 Patienten (durchschnittliches Alter zum Operationszeitpunkt 62 Jahre) nach einem durchschnittlichen Follow-up von 2,5 Jahren nachuntersucht. Der modified Harris Hip Score (mHHS) verbesserte sich von 48 Punkten präoperativ auf 60 Punkte zum Follow-up. Präoperativ wiesen 100 % ein positives Trendelenburg-Zeichen auf, zum Follow-up Zeitpunkt waren es ca. 50 %.

## Abstract (en)

**Objective:**

Transfer of the gluteus maximus with refixation at the greater trochanter for treatment of abductor deficiency.

**Indications:**

Symptomatic abductor deficiency with atrophy and fatty degeneration of the gluteal muscles > 50% (grade 3 by quartile) with good strength of the gluteus maximus.

**Contraindications:**

Low atrophy or fatty degeneration of less than 50% of the gluteal muscles, limited strength of the gluteus maximus, infection.

**Surgical technique:**

First, the fascia lata is incised dorsally to the tensor fascia latae muscle, with the incision extending approximately 1.5 cm proximal to the iliac crest. A second incision divides the gluteus maximus muscle longitudinally along the muscle fibers and continues towards the fascia lata distal to the greater trochanter. These incisions result in a triangular muscle flap, which is elevated and divided into anterior and posterior portions. The posterior flap is positioned ventrally over the femoral neck and fixed to the anterior capsule and the anterior edge of the greater trochanter. The anterior flap is placed directly on the proximal femur. For this purpose, a groove is prepared in the area of the proximal femur using a spherical burr to freshen up the future footprint. The anterior flap is positioned from the tip of the greater trochanter towards the insertion of the vastus lateralis muscle. Subsequently, the anterior flap is fixed to the created groove with transosseous sutures and positioned under the elevated vastus lateralis muscle in 15° abduction of the leg. To provide additional stabilization to the tendinous part of the anterior flap, a screw is inserted distally to the greater trochanter. The vastus lateralis muscle is attached to the distal tip of the anterior flap, and the remaining gluteus maximus muscle is sutured to the fascia lata to cover the anterior flap. Additionally, a flap of the tensor fascia latae muscle can be mobilized and adapted to the reconstruction. Layered wound closure is performed.

**Results:**

The technique of a gluteus maximus transfer represents a method for the treatment of chronic abductor deficiencies and improves abduction function as well as the gait pattern in short-term follow-ups. Fifteen patients (mean age at time of surgery 62 years) had after a mean follow-up of 2.5 years. The modified Harris Hip Score (mHHS) improved from 48 points preoperatively to 60 points at follow-up. Preoperatively, 100% had a positive Trendelenburg sign; at follow-up, this was about 50%.

## Vorbemerkungen

Eine Insuffizienz der glutealen Muskulatur, insbesondere der Mm. glutei medius und minimus, kann zu Schmerzen, Hinken und Funktionseinschränkungen führen [5, 18, 20]. Die häufigste Ursache für eine symptomatische Abduktorenschwäche ist eine iatrogene Läsion der Insertion der Mm. glutei medius und/oder minimus am Trochanter major im Rahmen von einer endoprothetischen Versorgung des Hüftgelenkes (v. a. bei Verwendung eines lateralen Zugangs) oder des N. gluteus superior [19], die zu einer fettigen Degeneration mit folgender Muskelschwäche führen kann [5, 18, 19]. Aber auch degenerative Veränderungen der Sehnen mit chronischer Tendinopathie oder folgenden Rupturen können ursächlich für eine gluteale Insuffizienz sein [2]. Typischerweise berichten die Patienten massivste peritrochantäre Schmerzen, die in Seitenlage unter Druck verstärkt werden können, wobei Frauen ab der 5. Lebensdekade bis zu 9‑mal häufiger betroffen sind als Männer [4, 8, 12, 13]. Entsprechend dem Ausmaß der Schädigung kann ergänzend ein belastungsabhängiges Insuffizienzhinken auftreten. Zudem kann auf der betroffenen Seite die Tendenz beobachtet werden, dass das Bein aufgrund einer verminderten Innenrotationskraft eine Außenrotationsstellung einnimmt. In erste Linie wird bei glutealen Insuffizienzen eine konservative Therapie angestrebt, die sowohl physiotherapeutische Beübung mit Kräftigung der hüftumgreifenden Muskulatur als auch die Einnahme von nichtsteroidale Antirheumatika (NSAR) sowie eine Anpassung des Lebensstils beinhaltet. Gegebenenfalls kann ergänzend eine transkutane elektronische Nervenstimulation (TENS) zur Anwendung kommen. Führt die konservative Therapie über einen Zeitraum von mindestens 3 bis 6 Monaten zu keiner merklichen Besserung, so sollte ein operatives Vorgehen diskutiert werden. In der Literatur sind hierfür diverse Techniken beschrieben: eine Refixation der Sehnen mit Knochenankern [15], eine Verlagerung des Vastus lateralis [1, 3, 17], ein Gluteus-maximus-Transfer [21, 22] oder eine Rekonstruktion mit Allografts [10, 14]. Die gewählte Operationsmethode und die Ergebnisse der chirurgischen Therapie hängen dabei von der Größe und Art der Muskelveränderung ab [19]. Beim Vorliegen einer großen Läsion mit deutlicher Verfettung der Mm. glutei medius und minimus hat sich in den letzten Jahren der Gluteus-maximus-Transfer als vielversprechender Lösungsansatz gezeigt [9, 19, 21, 22]. Der vorliegende Beitrag soll daher die (Kontra‑)Indikationen sowie die schrittweise Darstellung der operativen Technik eines Gluteus-maximus-Transfers zur Behandlung einer ausgeprägten glutealen Insuffizienz darstellen.

## Operationsprinzip und -ziel

Durchführung eines Transfers des M. gluteus maximus mit Refixation am Trochanter major zur Therapie der glutealen Insuffizienz.

## Vorteile

- Der Ursprung und der Ansatz der kleinen Abduktoren (Mm. glutei medius et minimus) bleiben intakt
- Durchführbar auch bei vorliegendem Knochendefekt
- Möglichkeit einer aktiven Abduktion bei unzureichender Kraftentwicklung der kleinen Abduktoren (Mm. glutei medius et minimus)

## Nachteile

- Anspruchsvolle Operationstechnik, die durch einen erfahrenen Chirurgen durchgeführt werden sollte
- Resultierender, abnormaler Kraftvektor für die Hüftabduktion
- Im Revisionsfall oftmals komplexer Situs aufgrund der extraanatomischen Veränderungen
- Reduktion der Kraftleistung und Funktion des M. gluteus maximus

## Indikationen

- Klinisch symptomatische gluteale Insuffizienz mit Atrophie und fettiger Degeneration der Glutealmuskeln > 50 %, Grad 3 nach Quartile [11]
- Ausgeschöpfte konservative Therapie (keine ausreichende Funktion von Gluteus medius et minimus nach mindestens 6‑monatiger erfolgloser Therapie)
- Gut erhaltene Kraft des M. gluteus maximus (> 3/5 nach Janda)

## Kontraindikationen

- Geringe Atrophie oder fettige Degeneration von weniger als 50 % der Glutealmuskulatur, sodass eventuell eine Refixation in Betracht gezogen werden kann
- Eingeschränkte Kraft des M. gluteus maximus (< 4/5 nach Janda)
- Infektionen
- Lokalisierte und generalisierte Entzündungen
- Gelenknahe Knochentumoren
- Therapiebedürftige Probleme einer einliegenden Hüftendoprothese (Fehllage, Lockerung, Offset, etc.)
- Neurochirurgisch rekonstruierbare Nervenläsion als Ursache für die Atrophie
- Fortgeschrittene Koxarthrose

## Patientenaufklärung

- Allgemeine Operationsrisiken wie Wundheilungsstörung, Infektion, Gefäß- und Nervenverletzungen, Blutung, Thrombose und Embolie
- Verbleib einer Narbe von ca. 15–20 cm Länge
- Persistenz der Schmerzen und des Hinkens
- Beschreibung des resultierenden Defizits der Funktion des Gluteus maximus
- Versagen der Rekonstruktion
- Postoperative Ossifikationen
- Postoperative Nachbehandlung (mehrwöchige Teilbelastung und Orthesenversorgung)
- Erfolgsaussicht ca. 50 %

## Operationsvorbereitungen

- Präoperativ sind eine gründliche Anamnese mit vorausgegangenen Operationen und eine körperliche Untersuchung obligatorisch. Die körperliche Untersuchung beinhaltet eine sorgfältige Beurteilung des Gangbildes, der Beckenstabilität inklusive des Trendelenburg-Zeichens sowie eine Kraftbeurteilung der Hüftabduktoren und des M. gluteus maximus.
- Anfertigung von Röntgenaufnahmen: korrekt eingestellte Beckenübersichtsaufnahme sowie das betroffene Hüftgelenk axial (Basisdiagnostik, Beurteilung einer etwaig einliegenden Hüftprothese, Ausmaß arthrotischer Veränderungen sowie der Trochanterregion).
- Außerdem ist eine hochauflösende Magnetresonanztomographie (MRT) zur Beurteilung der pelvitrochantären Muskulatur zwingend empfohlen. Hierbei hat sich folgender Algorithmus als hilfreich gezeigt [16]: Als Erstes sollten in der axialen T2-Sequenz die Größe des M. tensor fasciae latae (TFL), die anteriore, laterale und posterior-superiore Facette sowie ein möglicher Flüssigkeitssaum zwischen Sehne und Trochanterinsertion beurteilt werden. Im nächsten Schritt können in der koronaren T2-Sequenz die anterioren, lateralen und posterior-superioren Facetten, die Sehnenlänge, mögliche Unterseitenläsion sowie eine Bursabeteiligung analysiert werden. Als letzter Schritt empfiehlt sich eine Betrachtung der koronaren T1-Sequenzen, um erneut die Größe des TFLs, aber auch den Grad der Verfettung der pelvitrochantären Muskulatur beurteilen zu können. Die Autoren bevorzugen zur Gradierung der Verfettung die Quartile-Klassifikation ([11]; Tab. 1).

**Tab. 1:** Quartile-Klassifikation [11]

Gradeinteilung	Prozentualer Fettanteil
Grad 0	Normaler Muskel
Grad 1	Fettanteil 1–25 %
Grad 2	Fettanteil 26–50 %
Grad 3	Fettanteil 51–75 %
Grad 4	Fettanteil 76–100 %

## Instrumentarium

- Pinzetten und Skalpell
- Präparierschere
- Scharfe Haken
- Wundspreizer
- 2- und 3,2-mm-Bohrer
- 4,5-mm-Kortikalisschrauben mit Unterlegscheibe
- Hochgeschwindigkeitsfräse (Midas Rex Legend EHS Stylus, Medtronic, Dublin, Irland)
- Nahtmaterial (nicht-resorbierbar und resorbierbar)

## Anästhesie und Lagerung

- Allgemeinanästhesie oder Spinalanästhesie
- Perioperative Antibiotikaprophylaxe
- Seitenlagerung mit Unterpolsterung des zu operierenden Beines. Eine leichte Abduktion empfiehlt sich zur Entlastung der Glutealmuskulatur.

## Operationstechnik

**Abb. 1:**
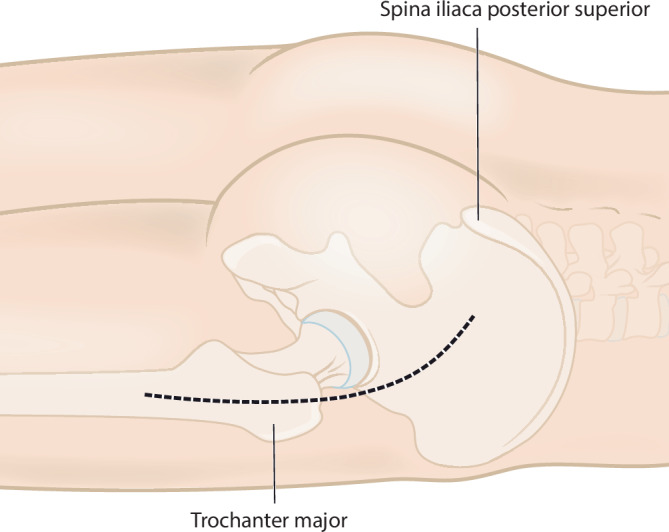
Als Zugangsweg wird ein posteriorer oder alternativ ein Kocher-Langenbeck-Zugang gewählt. Der Hautschnitt erfolgt in Längsrichtung über dem Trochanter major und verläuft kranial nach posterior abfallend in Richtung Crista iliaca

**Abb. 2:**
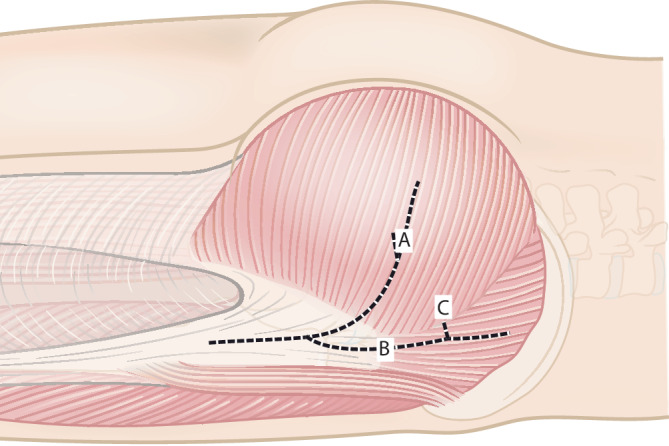
Subkutane Präparation und Darstellung des Tractus iliotibialis. Präparation eines dreieckigen Lappens des M. gluteus maximus. Hierzu erfolgt die Traktusinzision beginnend auf Höhe des proximalen Femurs in Femurlängsrichtung nach kranial, dann abfallend in Verlaufsrichtung der Fasern des M. gluteus maximus nach dorsal bis zur Hälfte des M. gluteus maximus als posteriore Grenze des Muskellappens (*A*). Der anteriore Anteil des Lappens ergibt sich aus der Verlängerung der Traktusinzision nach kranial in Faserlängsrichtung bis vor den Ansatz an der Crista iliaca, ca. 1–1,5 cm ventral des M. gluteus maximus (*B*) mit einer horizontalen Inzision bis zum ventralen Rand des Muskels (*C*)

**Abb. 3:**
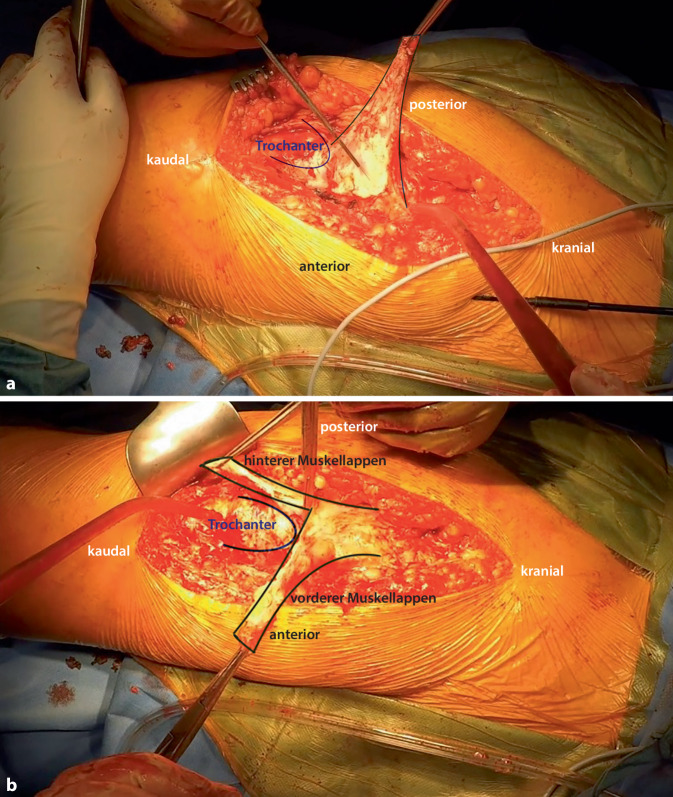
Der dreieckige Muskellappen wird angehoben (**a**) und in einen vorderen und einen hinteren Teil geteilt (**b**), sodass die jeweilige Breite der Basis ca. 4–5 cm beträgt. Anatomische Untersuchungen haben gezeigt, dass der Eintritt der neurovaskulären Strukturen ca. 2 cm vom Foramen ischiadicum majus entfernt liegt und eine Beeinträchtigung durch die beschriebene Lappenbildung nicht beeinflusst wird [22]

**Abb. 4:**
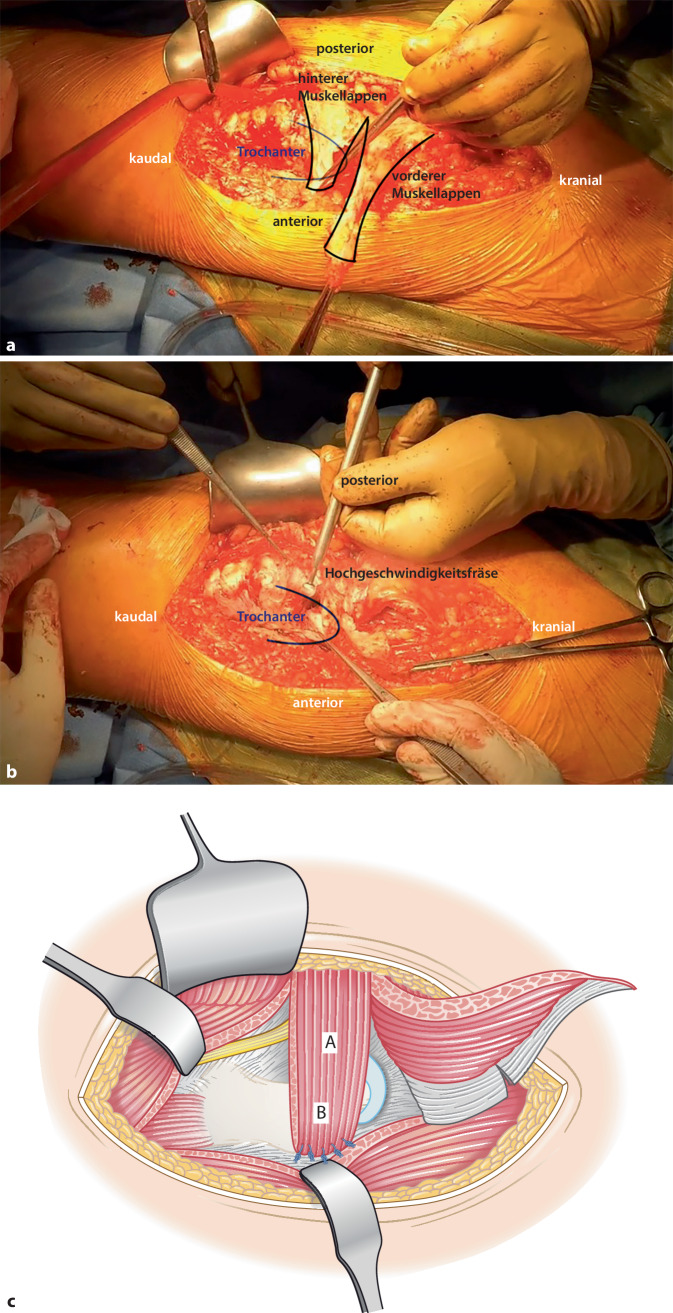
Der posteriore Anteil des Lappens wird über die Trochanterspitze nach anterior geschwenkt (**a**), bei welcher zuvor ein Sulcus mit einer Hochgeschwindigkeitsfräse (**b**) geschaffen wurde (bis zur Spongiosa), damit der Muskellappen einwachsen kann. Dann wird dieser mit den ventralen Kapselanteilen sowie in dem ventralen Anteil des Trochanter major vernäht (**c**)

**Abb. 5:**
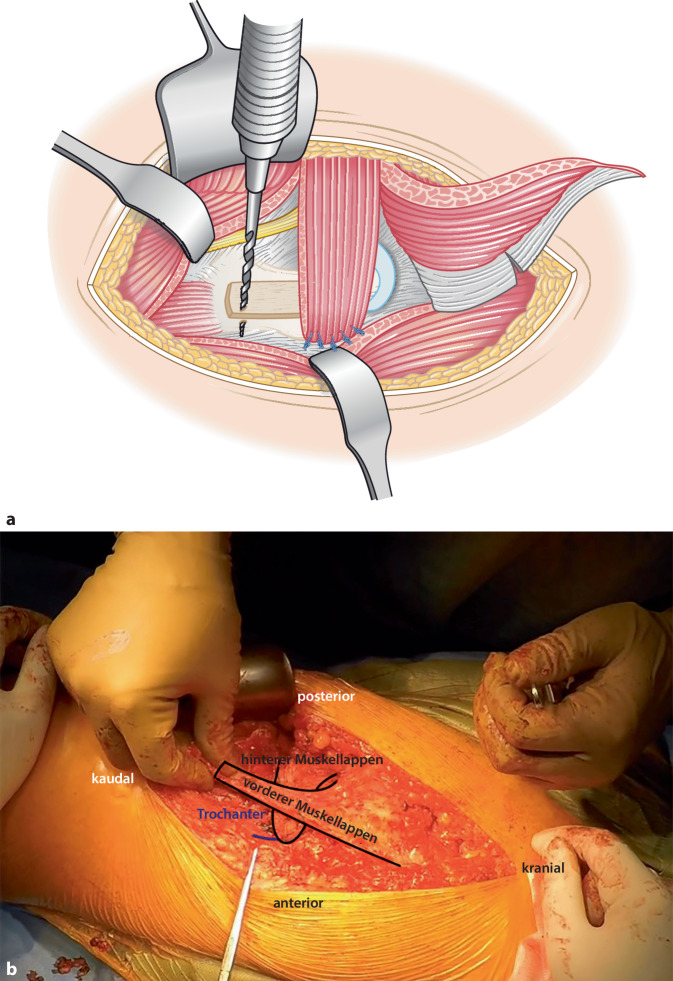
Nachdem auch im Bereich des lateralen proximalen Femurs mit der Hochgeschwindigkeitsfräse ein Sulcus gefräst wurde, werden in die Kortikalis mit einem 2‑mm-Bohrer sowohl nach ventral als auch nach dorsal gerichtet jeweils mehrere Löcher gebohrt (**a**). Durch die Bohrlöcher wird der anteriore Anteil des Muskellappens, welcher über den posterioren Anteil umgeschlagen wird, an das proximale Femur mit nichtresorbierbaren Fäden unter maximaler Vorspannung vernäht (**b**). Eine milde Abduktion von 15° kann hierbei hilfreich sein

**Abb. 6:**
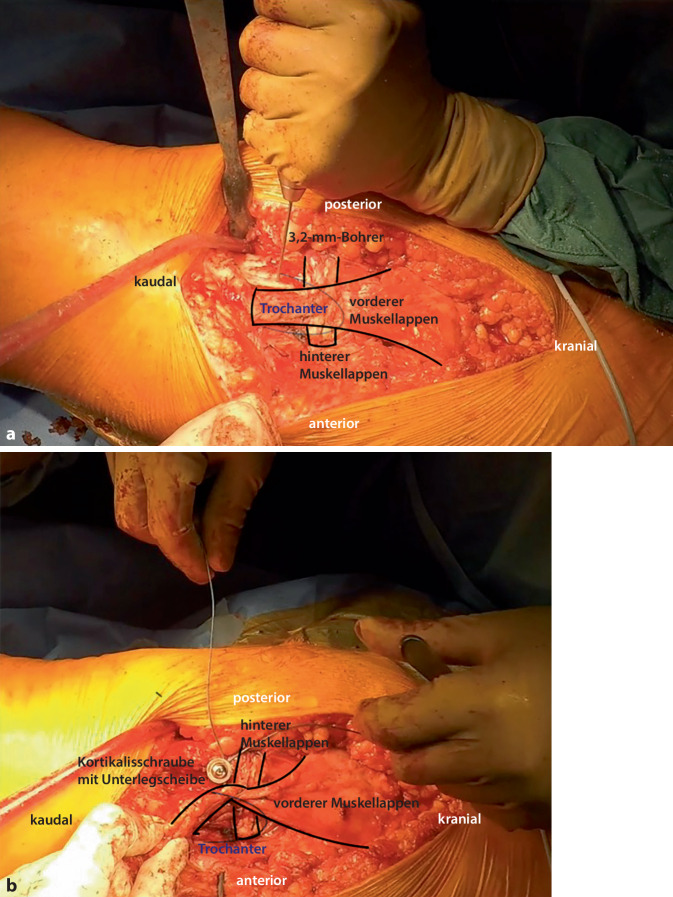
Als Modifikation der ursprünglich von Whiteside 2012 beschriebenen Technik [21] erfolgt ergänzend eine Fixierung des Muskellappens mit einer 4,5-mm-Kortikalisschraube mit Unterlegscheibe. Es wird mit einem 3,2-mm-Bohrer von lateral nach medial gebohrt (**a**). Der Lappen wird mit einem nichtresorbierbaren Faden armiert und dieser um die Schraube geschlungen sowie verknotet (**b**)

**Abb. 7:**
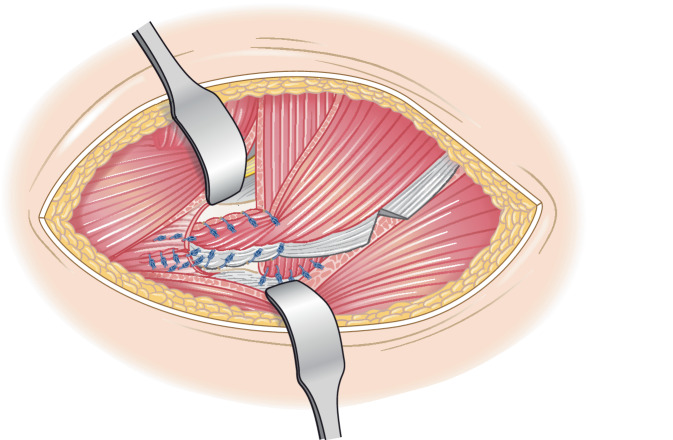
Der proximale Teil des M. vastus lateralis, der zuvor längs inzidiert wurde, wird nun auf den Muskellappen genäht und dieser additiv fixiert

(Abb. 1, 2, 3, 4, 5, 6, 7, 8 und 9)

**Abb. 8:**
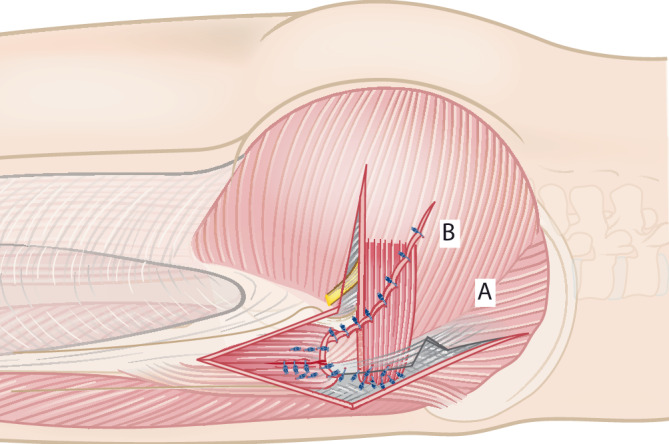
Der M.-gluteus-maximus-Lappen (*B*) wird mit dem posterioren Anteil des Tractus iliotibialis (*A*) vernäht und dann der distale Anteil des Traktus an den Vastus lateralis genäht

**Abb. 9:**
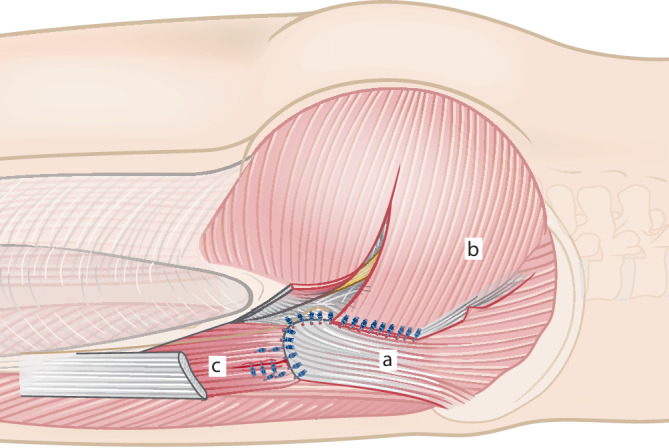
Als Modifikation der ursprünglichen von Whiteside 2012 beschriebenen Technik [21] kann ergänzend ein Transfer des M. tensor fasciae latae (*a*) erfolgen. Die Fascia lata wird transversal bis zum lateralen Rand des M. sartorius inzidiert, wobei eine mindestens 1 cm breite Fascia-lata-Manschette am distalen Ende des M. tensor fasciae latae verbleibt. Der M. tensor fasciae latae wird nach proximal vom M. sartorius getrennt. Der gewonnene Lappen des M. tensor fasciae latae wird über den Trochanter major und den M.-gluteus-maximus-Lappen verlagert und transossär fixiert. Das distale Ende kann nach posterior überlappt werden, um einen Kapseldefekt zu rekonstruieren. Abschließend erfolgt die Naht des Tractus iliotibialis mit schichtweisem Wundverschluss

## Postoperative Behandlung

- Als postoperative Ossifikationsprophylaxe wird ein NSAR unter Magenschutz für 21 Tage verabreicht. Bis zum Erreichen der Vollbelastung sollte eine Thromboembolieprophylaxe durchgeführt werden.
- Die postoperative Mobilisierung wird unter Teilbelastung von 15 kg mit Unterstützung von Unterarmgehstützen für 6 Wochen durchgeführt und bis zur 8. postoperativen Woche zur Vollbelastung fortgeführt.
- Versorgung der operierten Hüfte mit einer Hüftorthese für 6 Wochen (z. B. Hipocross-Orthese der Firma Orthoservice, Chiasso, Schweiz). Diese wird mit einer Limitierung der Flexion auf 90° eingestellt.
- Eine aktive Abduktion und Außenrotation sollten für 6 Wochen konsequent vermieden werden.
- Nach der 6. postoperativen Woche kann mit physiotherapeutischer Beübung begonnen werden, wobei die Abduktion in der 7. und 8. postoperativen Woche passiv assistiert und ab der 9. Woche gegen die Schwerkraft beübt werden sollte. Eine Gangschule und intensiveres Training der Abduktion gegen Widerstand werden 3 Monate postoperativ begonnen.

## Fehler, Gefahren, Komplikationen

- Gefahr des Nahtversagens: Um der Gefahr eines Nahtversagens entgegenzuwirken, empfehlen die Autoren die additive ossäre Fixierung mit einer Schraube inklusive Unterlegscheibe. Zusätzlich sollten eine aktive Abduktion und Außenrotation für mindestens 6 Wochen vermieden werden. Hier ist eine präoperative Aufklärung des Patienten zwingend erforderlich, um die Wichtigkeit der Nachbehandlung zu betonen und die Compliance zu erhöhen. Sollte es zu einem Nahtversagen kommen, könnte ein monofilamentäres Polypropylen-Netz (z. B. Marlex-Mesh, Fa. Bard, Murray Hill, USA) verstärkend eingelegt werden.
- Gefahr eines zu dünnen Sehnenlappens: Um der Gefahr eines zu dünnen Sehnenlappens entgegenzuwirken, empfiehlt es sich, eine ausreichende Breite des Lappens von mindestens 2 cm zu wählen, damit eine ausreichende Refixation der einzelnen Zügel erreicht werden kann.
- Gefahr einer zu geringen Mobilisierung des anterioren Lappens: Um eine ausreichende Mobilisierung des anterioren Lappens gewährleisten zu können, empfiehlt es sich, den Lappen an der anterioren Begrenzung horizontal zu inzidieren (s. mit *C* bezeichnete Inzision in Abb. 2).
- Individuell zu schmaler Tractus iliotibialis: Sollte aufgrund der individuellen Anatomie ein schmal ausgeprägter Tractus iliotibialis vorliegen, so kann ergänzend der M. tensor fasciae latae mobilisiert und auf das Rekonstruktionskonstrukt genäht werden.
- Knöcherner Defekt des proximalen Femurs: Im Falle eines knöchernen Defektes des proximalen Femurs kann eine additive Refixation mit einer Drahtcerclage anstatt der beschriebenen Schraube erfolgen.

## Ergebnisse

Die im vorliegenden Artikel beschriebene Technik stellt dabei eine Salvage-Methode dar, um chronische gluteale Insuffizienzen im Endstadium zu behandeln. Erstmalig wurde diese Technik durch Whiteside im Jahr 2012 beschrieben [21]. Der Vorteil liegt darin, dass die anatomische Position sowie die ursprüngliche Funktion und die Richtung der Muskelfasern des M. gluteus maximus mit denen der insuffizienten kleinen Abduktoren übereinstimmen. Die Lage der neurovaskuläre Versorgung [22] erleichtert, wie oben beschrieben, den Transfer dieser Muskeln auf den Trochanter major, ohne die Kraft im Bereich der Entnahmestelle zu beeinträchtigen. Whiteside berichtet dabei gute Ergebnisse mit fehlendem Trendelenburg-Zeichen bei ca. 80 % der versorgten Patienten und normalisiertem Gangbild 2 bis 5 Jahre postoperativ [22, 23]. Als Modifikation der ursprünglich beschriebenen Technik kann zusätzlich der M. tensor fasciae latae auf den Trochanter major geschwenkt und fixiert werden. Die Kombination aus beiden Muskeln verbesserte dabei in kurzzeitigen Follow-ups die Abduktionsfunktion und -kraft der Hüfte mit normalisiertem Gangbild und reduzierte die Schmerzen mit zufriedenstellenden Ergebnissen bei > 80 % der Patienten [6, 7].

In der vorliegenden Arbeit präsentieren wir die ersten 15 Patienten (13 Frauen und 2 Männer), die vom Seniorautor zwischen 2018 und 2022 mit dieser Technik operiert wurden, wobei 5/15 Patienten einen Transfer des M. tensor fasciae latae erhielten. Bei 13 Patienten bestand ein Zustand nach Hüft-Totalendoprothesen-Implantation über einen lateralen Zugang, bei 2 Patienten lag eine schwere Degeneration der Gluteus-medius- und -minimus-Muskulatur mit resultierender glutealer Insuffizienz vor. Die präoperative MRT-Diagnostik ergab bei 9 Patienten eine Degeneration Grad 3 und bei 6 Patienten eine Degeneration Grad 4 nach Quartile der Glutei medius und minimus. Das mittlere Follow-up betrug 2,5 (1 bis 4,5) Jahre. Das durchschnittliche Alter zum Operationszeitpunkt lag bei 62 (55 bis 79) Jahre und der durchschnittliche Body Mass Index (BMI) bei 27 (21–37) kg/m^2^. Die durchschnittliche Operationszeit betrug 134 min. Der Schmerz anhand visueller Analogskala (VAS) konnte von 6 (1–10) auf 4 (0–8) gesenkt werden (*p* = 0,17). Im modified Harris Hip Score (mHHS) zeigte sich ebenfalls eine Verbesserung von 48 (25–92) auf 60 (30 bis 100) Punkte (*p* = 0,24). Während präoperativ bei 100 % der Patienten ein positives Trendelenburg-Zeichen vorlag, präsentierten sich 47 % der Patienten zum Follow-up-Zeitpunkt mit einem negativen Trendelenburg-Zeichen. Die Abduktionskraft nach Janda konnte von präoperativ 2,5 (1 bis 5) auf 3,0 (2 bis 5) Punkte verbessert werden (*p* = 0,29). Die Kraft des M. gluteus maximus lag prä- sowie postoperativ bei allen Patienten > 3 und zeigte sich folglich nicht reduziert.

Die in der Literatur beschriebenen sehr guten Ergebnisse konnten durch die vorliegenden eigenen Daten nicht bestätigt werden. Neben kleinen Studienkohorten wurden die sehr guten Literaturergebnisse in Patientenkollektiven mit einem deutlich geringeren präoperativen Grad an Muskeldegeneration und Abduktoreninsuffizienz erhoben.

Die exakte präoperative klinische als auch MRT-morphologische Evaluation der pelvitrochantären Muskulatur ist daher von entscheidender Bedeutung. Eine ausführliche Aufklärung über das zu erwartende limitierte postoperative funktionelle Outcome muss bei Patienten mit fortgeschrittener Muskeldegeneration und Abduktoreninsuffizienz erfolgen. Es ist zu beachten, dass diese Patienten häufig langwierige und frustrane konservative Therapien durchlebt haben und das Ziel des operativen Vorgehens die Prävention einer weiteren Verschlechterung der Symptome und Funktion ist.

Zusammenfassend stellt die gezeigte Technik eines M.-gluteus-maximus-Transfers in etwa der Hälfte eine Salvage-Methode zur Behandlung chronischer glutealer Insuffizienzen dar, wobei Langzeitdaten bisher noch ausstehend sind.
